# Splitting in Schizophrenia and Borderline Personality Disorder

**DOI:** 10.1371/journal.pone.0091228

**Published:** 2014-03-06

**Authors:** Ondrej Pec, Petr Bob, Jiri Raboch

**Affiliations:** 1 Center for Neuropsychiatric Research of Traumatic Stress, Department of Psychiatry, First Faculty of Medicine, Charles University, Prague, Czech Republic; 2 Psychotherapeutic and Psychosomatic Clinic ESET, Prague, Czech Republic; 3 Central European Institute of Technology, Faculty of Medicine, Masaryk University, Brno, Czech Republic; Catholic University of Sacred Heart of Rome, Italy

## Abstract

**Background:**

Splitting describes fragmentation of conscious experience that may occur in various psychiatric disorders. A purpose of this study is to examine relationships between psychological process of splitting and disturbed cognitive and affective functions in schizophrenia and borderline personality disorder (BPD).

**Methods:**

In the clinical study, we have assessed 30 patients with schizophrenia and 35 patients with BPD. The symptoms of splitting were measured using self-reported Splitting Index (SI). As a measure of semantic memory disorganization we have used verbal fluency test. Other psychopathological symptoms were assessed using Health of the Nation Outcome Scale (HoNOS).

**Results:**

Main results show that SI is significantly higher in BPD group than in schizophrenia, and on the other hand, verbal fluency is significantly lower in schizophrenia group. Psychopathological symptoms measured by HoNOS are significantly higher in the BPD group than in schizophrenia. Significant relationship was found between verbal fluency and the SI “factor of others” (Spearman r = −0.52, p<0.01) in schizophrenia patients.

**Conclusions:**

Processes of splitting are different in schizophrenia and BPD. In BPD patients splitting results to mental instability, whereas in schizophrenia the mental fragmentation leads to splitting of associations observed as lower scores of verbal fluency, which in principle is in agreement with Bleuler’s historical concept of splitting in schizophrenia.

## Introduction

Splitting reflects shifts of mind related to a consciously experienced conflict of opposing mental forces. In principle it describes fragmentation of conscious experience that is typically related to long-term or acute stress that significantly disturbs self-concept, identity, memory and perception of the external world [1]–[5]. Nevertheless, empirical studies of psychopathological processes related to splitting are very rare.

In schizophrenia the term splitting was developed by Bleuler [6], who described process of mental fragmentation in schizophrenia as associative splitting or “loosening of associations” and considered it as a basic factor in pathogenesis of the disease. Later concept of splitting was described by Kernberg [7], who used the process of splitting as a specific characteristic of cognitive and affective disturbances in borderline personality disorder (BPD) which typically manifest as shifts of emotional perception of objects, other persons and the self with typical fluctuations between idealization and devaluation.

These alterations on mental level consequently may be linked to great and abrupt changes in patterns of neural activity that may dissociate, or split off, certain external and internal stimuli and information out of awareness, which may lead to distinct states of divided consciousness [4], [8]–[11] and disorganization of semantic memory [12]–[14].

With respect to recent findings a purpose of this study is to examine relationships between psychological process of splitting and disturbed cognitive and affective functions in schizophrenia and BPD.

## Materials and Methods

### Participants

The participants were recruited from regular daily outpatients treatment programs for schizophrenic and BPD patients at the Psychotherapeutic and Psychosomatic Clinic ESET in Prague. All participants signed informed consent and the study was approved by Charles University ethical committee. In the study were included only patients who had not compromised capacity and ability to consent. This ability was confirmed by clinical data about the patients and specific written statement regarding each participant by his/her psychiatrist. Each included participant was able to consider his/her participation and no one was included in the study based on agreement of legally authorized representative consented on the behalf of a participant.

The participants had diagnosis of schizophrenia or borderline personality disorder. Exclusion criteria were organic illnesses involving the central nervous system, substance, and/or alcohol abuse and mental retardation (IQ Raven lower than 90) [15]. Clinical diagnosis was reassessed using the Mini-International Neuropsychiatric Interview (M.I.N.I.) [16] in schizophrenia patients and in BPD patients it was confirmed using semi-structured interview for borderline personality disorder based on DSM-IV criteria. The sample included 30 patients with schizophrenia, i.e. 15 men and 15 women, mean age 35.7 (SD = 9.2) with mean period of psychiatric treatment 12.89 (SD = 7.8 years) and with average of 4.1 hospitalizations. The sample of BPD patients included 35 participants, i.e. 10 men and 25 women, mean age 32.0 (SD = 7.9) years with mean period of psychiatric treatment 6.2 (SD = 3.97) years and with average of 2.28 hospitalizations.

### Psychometric Measures

With respect to current theoretical concepts and empirical data we have tested relationship between splitting based on Splitting Index score [17] and verbal fluency as an indicator of semantic memory disorganization [12]–[13], [18] in patients with schizophrenia and BPD. To test how the splitting process is typically represented in schizophrenia and BPD we have compared occurrence of these psychopathological manifestations in schizophrenia and BPD and their relationships to other symptoms.

The symptoms of splitting were measured using self-reported Splitting index (SI) [18] that enables to assess defense mechanisms related to splitting according to concept proposed by Kernberg [7]. Splitting Index is 24-items self-reported questionnaire rated on 5-point Likert scale from 1 to 5 (Cronbach’s alfa 0.92, test-retest reliability after one week 0.82). Using factor analysis three clusters of items have been identified that enable to describe the splitting process. These three factors represent: 1. the self factor (splitting of the self image), 2. the family factor (splitting of images of family members), and 3. the factor of others which describes splitting with respect to people outside the family.

Other psychopathological manifestations in both groups of patients were measured using Health of the Nation Outcome Scales (HoNOS) [19]. The scale includes 12 items including overactive, aggressive, disruptive or agitated behavior; non-accidental self-injury; problem drinking or drug-taking; cognitive problems; physical illness or disability problems; hallucinations or delusions; problems with depressed mood; other mental and behavioral problems; problems with relationships; problems with activities of daily living; problems with living conditions; problems with occupation and activities. This scale includes two versions, i.e. the version for external evaluators and the self-reported version (Cronbach’s alfa 0.79, test-retest reliability after one week 0.85) [20].

As a measure of semantic memory disorganization, which is very close to Bleuler’s concept of mental fragmentation, we have used verbal fluency test [12]–[13], [21]. In this context, recent findings show that verbal fluency is severely disturbed in schizophrenia [22] and it is closely related to disorganized dimension of psychopathology in schizophrenic patients [14].

### Data Analysis

Statistical evaluation of the results of SI and other psychometric measures included descriptive statistics, Mann-Whitney test for independent samples and Spearman correlation coefficients. The non-parametric analyses were preferred because SI data have not normal distribution. All the methods of statistical evaluation were performed using the software package Statistica version 6. To prevent Type II error which would disable to reject null hypothesis that the measure of splitting is not linked to verbal fluency and psychopathological symptoms we performed Power Analysis and assessed the effect sizes characterizing differences between means and correlation coefficients.

## Results

Results show significant differences in scores of splitting, verbal fluency and psychopathological symptoms measured by HoNOS between BPD and schizophrenia groups that were compared using Man-Whitney test (Table 1). Mean score of the Splitting Index (SI) was significantly higher in BPD group than in schizophrenia.

**Table 1:** Statistical comparison between schizophrenia and BPD patients using Mann-Whitney test.

	Schizophrenia N = 30	BPD N = 35	Z	p	R
**SI**	2.84	3.14	−2.2	0.0025	0.58
**SI(S)**	2.69	3.43	−2.8	0.0053	0.82
**SI(F)**	2.87	3.01	−1.0	0.3384	0.14
**SI(O)**	2.96	2.99	−0.2	0.8759	0.05
**VF**	34.5	41.43	−2.8	0.0052	0.79
**HoNOS (E)**	11.6	15	−2.6	0.0085	0.81
**HoNOS (S)**	7.8	14.25	−3.9	0.0000	0.97

*Note:* SI – Splitting Index, SI(S) – Splitting Index, factor of self, SI(F) – Splitting Index, factor of family, SI(O) – Splitting Index, factor of others, VF- verbal fluency, HoNOS(E)- version for external evaluation of HoNOS (mean), HoNOS(S) - self-reported version of HoNOS (mean), BPD – borderline personality disorder, Z- Z value of Mann-Whitney test, r- standardized effect size.

On the other hand score of verbal fluency was significantly lower in schizophrenia group. In both assessments of HoNOS, for external evaluators and for self evaluation, the BPD group scored significantly higher in means of total scores. In the power analysis we have tested significant differences which show that all differences between means have strong effect size (r = 0.5 or higher; Table 1).

Results also show significant Spearman correlation coefficients characterizing relationships between splitting, verbal fluency and psychopathological symptoms measured by HoNOS in both samples (Table 2). Very significant relationship between verbal fluency and the SI “factor of others” in schizophrenia patients was found (Spearman r = −0.52, p<0.01). Other significant correlations in schizophrenia patients were found between self-reported score of HoNOS(S) and total score of splitting (SI) (Spearman r = 0.42, p<0.05) and between HoNOS(S) and SI(S) [representing splitting of the self] (Spearman r = 0.63, p<0.01). On the other hand significant correlations in borderline personality disorder were found between and between HoNOS(S) and SI(S) [representing splitting of the self] (Spearman r = 0.45, p<0.01) and HoNOS self-reported score and verbal fluency in has been found (Spearman r = 0.37, p<0.01).

**Table 2:** Spearman correlation coefficients between SI, verbal fluency, and HoNOS in schizophrenia and BPD patients.

	SI		SI-S		SI-F		SI-O		VF		HoNOS(E)	
	Sch	BPD	Sch	BPD	Sch	BPD	Sch	BPD	Sch	BPD	Sch	BPD
**SI–S**	**0.76**	**0.68**	_	_								
**SI-F**	**0.72**	**0.53**	**0.40**	0,05	_	_						
**SI–O**	**0.73**	**0.47**	0.27	0.01	**0.50**	0.09	_	_				
**VF**	−0.31	0.15	0.02	0.19	−0,23	0.05	**−0.52**	−0.05	_	_		
**HoNOS(E)**	0.26	0.13	0.28	0.33	0.21	0.14	0.09	−0.07	−0.22	0.15	_	_
**HoNOS(S)**	**0.42**	0.28	**0.63**	**0.45**	0.14	0,10	0.10	−0.10	0.16	**0.37**	**0.48**	**0.70**

*Note*: Values of Spearman correlation coefficients significant at p<0.05 are in bold (0.45 or higher are significant at p<0.01); Fisher Z was higher than 0.05; Sch – schizophrenia; BPD – borderline personality disorder; SI – Splitting Index; SI(S) – Splitting Index, factor of self; SI(F) – Splitting Index, factor of family; SI(O) – Splitting Index, factor of others; VF- verbal fluency; HoNOS(E): version for external evaluation; HoNOS(S): self-reported version.

## Discussion

Main results of this study indicate significant differences in splitting, verbal fluency and psychopathological symptoms between schizophrenia and BPD patients. These findings show significantly higher level of splitting measured by SI in BPD patients compared to schizophrenia. On the other hand schizophrenia patients show significantly lower scores of verbal fluency most likely as a consequence of cognitive disorganization which in principle is in agreement with Bleuler’s historical concept of splitting in schizophrenia [6]. In this context, the correlation between verbal fluency and splitting (factor of others) in schizophrenia suggests that stronger levels of splitting into opposite aspects related to external objects and persons is related to dis-association of memory patterns that is manifested as disturbed verbal fluency.

Recent findings also show that impaired verbal fluency is associated with psychomotor slowness [23]–[24] that might be related to disconnection between brain regions [24]. The disconnection between brain regions also disables integrated response to emotional stimuli, which might be linked to specific differences in amygdala activity and prefrontal functions in schizophrenia and BPD [25]. Schizophrenia is typically characterized by reduced activation in amygdala and prefrontal cortex and on the other hand increased and excessive activation in amygdala and prefrontal cortex has been found during emotional tasks in BPD [25]–[29].

These typical neurophysiological changes might reflect typical differences related to splitting in schizophrenia and BPD based on psychological mechanisms of defense against unacceptable affective impulses [30]. Responses to these impulses in BPD likely reflect disturbed levels of reality testing in response to various perceptual stimuli that typically result to increased and excessive emotional activation and irritability [3], [31]–[32], which on neurophysiological level could be reflected in increased prefrontal and amygdala activation [25]. On the other hand disturbed verbal fluency in schizophrenia patients is likely related to decreased activity in amygdala and prefrontal activity in schizophrenia [25]–[27], which might reflect inability to appropriately differentiate and reflect emotional stimuli [33]–[34] likely due to disruption in attentional selection and decision related activation [35].

In summary, the results show that the process of splitting has different forms in schizophrenia and BPD. In BPD patients splitting results to mental instability manifested as shifts in emotional perception of objects, other persons and the self, which are linked to increased mental tension and excessive prefrontal and amygdala activation. This specific form of splitting that occur in BPD is not typically present in schizophrenic patients, which is in agreement with the results indicating that SI score as a measure of borderline splitting is higher in BPD than in schizophrenia patients. On the other hand in schizophrenia the mental fragmentation leads to splitting of associations observed as lower scores of verbal fluency which in principle is in agreement with Bleuler’s historical concept of splitting in schizophrenia [35]. This form of mental fragmentation in schizophrenia may represent a defense mechanism decreasing several psychopathological manifestations due to lowered mental tension and abnormally inhibited brain activities in amygdala and prefrontal cortices. Nevertheless it is also possible that mental fragmentation in schizophrenic patients is also related to deficits in contextual processing that may be primarily based on brain’s ability to integrate information [35], [36]. This brain deficit to integrate information may be linked to various etiological conditions reflecting pathological processes on molecular, physiological and psychological levels. This brain potentiality to integrate information is on cognitive level specifically linked to ability to create integrated self-concept and synthetic capabilities related to various forms of metacognitive deficits that is typical impaired in schizophrenia [37]–[43].
